# Characterizing Mechanical Properties of Layered Engineered Wood Using Guided Waves and Genetic Algorithm

**DOI:** 10.3390/s23229184

**Published:** 2023-11-14

**Authors:** Nemish Atreya, Pai Wang, Xuan Zhu

**Affiliations:** 1 Department of Civil and Environmental Engineering, University of Utah, Salt Lake City, UT 84112, USA; 2 Department of Mechanical Engineering, University of Utah, Salt Lake City, UT 84112, USA

**Keywords:** engineered wood, mass timber, nondestructive evaluation, guided waves, numerical simulation, optimization, experimental mechanics

## Abstract

This study develops a framework for determining the material parameters of layered engineered wood in a nondestructive manner. The motivation lies in enhancing nondestructive evaluation (NDE) and quality assurance (QA) for engineered wood or mass timber, promising construction materials for sustainable and resilient civil structures. The study employs static compression tests, guided wave measurements, and a genetic algorithm (GA) to solve the inverse problem of determining the mechanical properties of a laminated veneer lumber (LVL) bar. Miniature LVL samples are subjected to compression tests to derive the elastic moduli and Poisson’s ratios. Due to the intrinsic heterogeneity, the destructive compression tests yield large coefficients of variances ranging from 2.5 to 73.2%. Dispersion relations are obtained from spatial–temporal sampling of dynamic responses of the LVL bar. The GA pinpoints optimal mechanical properties by updating orthotropic elastic constants of the LVL material, and thereby dispersion curves, in a COMSOL simulation in accordance with experimental dispersion relations. The proposed framework can support estimation accuracy with errors less than 10% for most elastic constants. Focusing on vertical flexural modes, the estimated elastic constants generally resemble reference values from compression tests. This is the first study that evaluates the feasibility of using guided waves and multi-variable optimization to gauge the mechanical traits of LVL and establishes the foundation for further advances in the study of layered engineered wood structures.

## 1. Introduction

Engineered wood or mass timber products have been gaining prominence in the construction industry [1]. The resilience of tall timber buildings recently became self-evident through a series of shake table tests on a 10-story timber building, which withstood a simulated 7.7 earthquake without major structural damages [2]. Compared with conventional solid wood, engineered wood offers greater design flexibility on the dimensions of manufactured structural components [3]. Moreover, as alternative construction materials to steel and concrete, mass timber products feature a higher strength-to-weight ratio [1,4] with a reduced carbon footprint. Layered engineered wood, including cross-laminated timber (CLT), mass ply panel (MPP), laminated veneer lumber (LVL), and glue-laminated timber (glulam), is among the most important and popular mass timber product category designs. With the increasing adoption of mass timber products, there is a potential rising demand for nondestructive evaluation (NDE) and quality assurance (QA) on layered engineered wood-based structural components, which have been challenging tasks due to their intrinsic structural complexity and heterogeneity.

Take the laminated veneer lumber or LVL as an example. LVL has been adopted in the design and construction of beams, columns, walls, and bridge decks [4,5,6,7,8] for its high strength and reliable mechanical properties. During its production, natural wood logs are cut into thin slices (veneers), dried, coated with adhesive, stacked together, and finally hot pressed to form a solid sheet. Natural wood has cellulose fibers in their natural alignment along the longitudinal direction (long grains); the radial direction goes from the center of the wood towards the bark; and the tangential direction is around the circumference of the log. LVL’s material properties, such as strength, stiffness, and thermal expansion, can vary significantly depending on the direction in which they are measured. Specifically, we assumed that LVL and layered engineered wood, in general, can be effectively modeled as an orthotropic material with nine independent elastic constants [9,10]. Furthermore, layered engineered wood shares origins with natural wood, and it is not without imperfections, including delamination, knots, and fiber defects, leading to variations in strength parameters [4,11]. Understanding the mechanical properties of layered engineered wood is key to material behavior prediction and design [12], but it is often challenging and limited by data scarcity [4].

Many research efforts have been invested on destructive and nondestructive tests for determining the elastic constants of solid woods [13,14,15], which could be applicable to layered engineered wood materials. For instance, compression tests on Scots pine wood yielded a 6 × 6 matrix of elastic constants with a 39% coefficient of variance for the shear modulus [13]. Similar studies on Eucalyptus Globulus wood also showed considerable variations, where the ultrasound tests provided parameter estimations closest to ground truth [14]. Dackermann et al. [15] employed static and ultrasound velocity testing to determine elastic constants in all directions. While elastic moduli along the longitudinal direction showed a reasonable agreement, measured values of Poisson’s ratio exhibited a 31% coefficient of variance, highlighting the challenges of measurement. Aira et al. [13] pointed out that ultrasound velocity testing is generally favored for its efficiency over laborious static bending and structural testing, and compression tests could lead to large variations (coefficient of variance or CoV) in the measured mechanical properties of wood materials. Therefore, alternative NDE methods for characterizing mechanical properties in orthotropic composites are reviewed, which can be applicable to engineered wood. Impulse- or impact-based vibration testing [16], exemplified by Gibson [17], utilizes broadband impulse vibration to determine elastic moduli and damping factors in fiber composite polymer materials. Considering multiple vibrational modes improved the accuracy of estimating elastic constants. Vibration testing also has the potential for detecting defects through natural frequency shifts. A typical vibrational test involves capturing the first five natural frequencies using three accelerometers and the Fast Fourier Transform (FFT) [17]. Researchers have also demonstrated the effectiveness of scanning laser Doppler vibrometry (SLDV) [18] on inspecting inlaid wood and easel paintings [19] based on vibration and ultrasound. Moreover, Josifovski et al. [20] reported the feasibility of using ultrasound and X-rays for in situ evaluation of timber structures. On the other hand, guided wave-based approaches provide another avenue whose analytical models, though complex, can describe wave propagation in anisotropic materials [21]. Morandi et al. [22] demonstrated that it is feasible to generate and measure guided wave dispersion in cross-laminated timber plates, where a bending mode was clearly identified.

Integrating experimental guided wave measurements with numerical models and optimization techniques is crucial for solving inverse problems and determining the mechanical properties of complex waveguides [23]. Both 2D and 3D finite element methods offer solutions for guided wave propagation [24,25,26]. Galerkin’s principle and energy velocities have been employed, revealing forward and backward waves in composite materials [27]. Fully discretized 3D finite element models were developed to understand dispersive guided wave propagation through commercial FEM software, such as Ansys 5.3 [28]. Furthermore, by assuming an analytical formulation along the wave propagation direction and only fully discretizing the 2D cross-section of a waveguide, the Semi-Analytical Finite Element (SAFE) method lends itself to compute wave dispersions with greatly reduced computational expenses [29,30,31]. More recently, researchers developed an extremely powerful tool that can be easily implemented in commercial FEM software for wave dispersion calculation leveraging Floquet periodicity [32]. Moreover, optimization algorithms have been developed for material characterization based on guided wave models and measurements. Zhu et al. [23] utilized an improved genetic algorithm (GA) to predict material properties, emphasizing the importance of proper wave mode selection for optimization. Their study achieved material property estimation accuracy of under 1%. Similar experiments involved composite plates, employing non-linear optimization to estimate material properties, achieving accuracy within a 10% range [27]. Cui et al. [29] used simulated annealing for anisotropic and quasi-isotropic laminates and analyzed wave modal sensitivities. Rautela et al. [33] developed a 1D Convolutional Neural Network for composite material properties, maintaining accuracy at around 1% with rapid training. Vishnuvardhan et al. [34] optimized all nine elastic constants of graphite–epoxy composite plates via GA, highlighting sensitivity analysis and achieving promising errors of approximately 1%. Genetic algorithms can generally support a performance similar to particle swarm optimization and outperform simulated annealing [35], and they are capable of handling complex multi-dimensional and multi-modal inverse problems [36]. Guided wave measurements incorporating computational optimization through GA offer precise material parameter estimation with enhanced accuracy and consistency compared to destructive testing, which enables material characterization and structural condition assessment. 

Prior research mainly focused on using destructive tests and computational simulations to characterize the mechanical properties of natural wood [13,14,15,37], identifying wave dispersion in mass timber plates [22], and structural health monitoring based on hygeothermal, static, and dynamic behavior of mass-timber buildings [38]. In this study, the very first framework to determine orthotropic elastic constants of layered engineered wood based on guided wave dispersion was proposed. Its feasibility was evaluated by determining orthotropic elastic constants of an LVL bar in a nondestructive manner. We acquired experimental dispersion curve data from the LVL bar, developed finite element models to understand the specific wave propagation behavior, and employed a genetic algorithm to optimize elastic constants by bridging experimental and computational approaches. This approach enhances predictive accuracy for engineered wood behavior, benefiting material scientists, engineers, and industry stakeholders. It promotes efficient and sustainable use of engineered wood, contributing to green and resilient structures.

## 2. Materials and Methods

**Figure 1:**
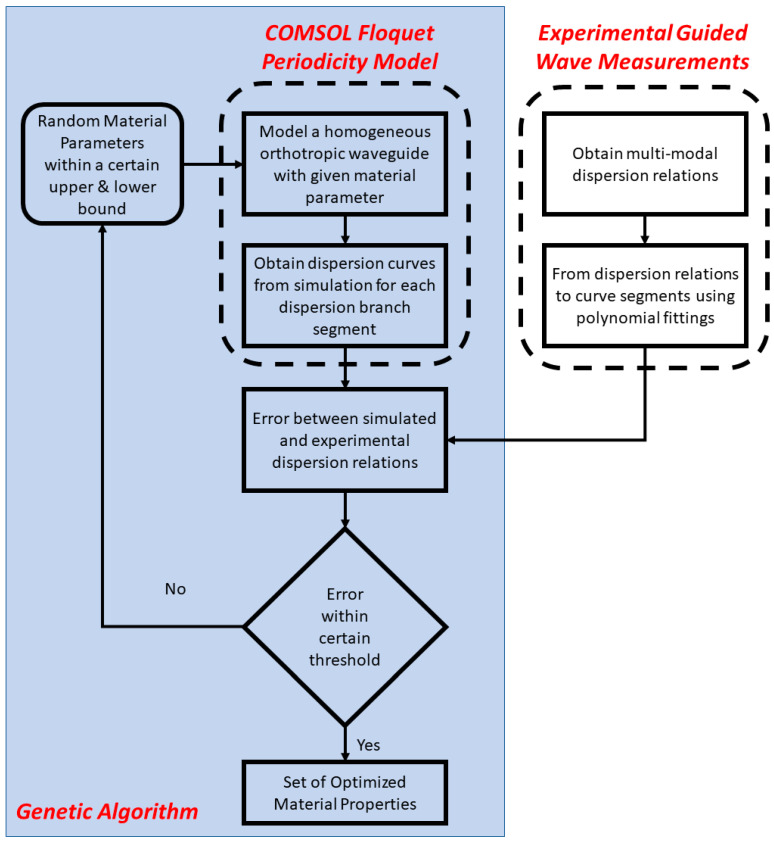
The proposed framework for estimating the mechanical properties of layered engineered wood.

This section explains the procedure of experimental testing (static compressive test and guided wave measurements), numerical modeling (Floquet periodicity model and frequency-domain analysis), and optimization via GA, along with their implementations and parallelization. The overview of the methodology is described in Figure 1. The key assumptions of this framework include that (i) one can experimentally recover multi-modal dispersion relations from a layered engineered wood product, and (ii) it is reasonable to model the LVL bar as a homogeneous orthotropic waveguide for guided wave propagation. 

### 2.1. Static Compression Test

**Figure 2:**
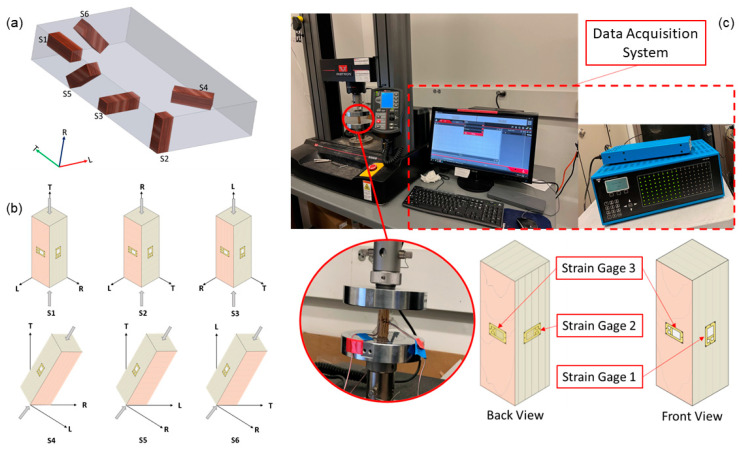
Static compression tests. (**a**) Sample configurations S1–S6 according to the grain direction. (**b**) Test samples and instrumentation. The axis shows the sample orientation, with each sample having an extra horizontally aligned strain gauge on the other side. (**c**) Data acquisition system setup for compression tests.

A series of compression tests was performed on cuboid-shaped samples extracted from an LVL bar (RigidLam Douglas-fir LVL APA 2.1E-3100Fb, Roseburg Forest Products, Springfield, OR, USA). This type of LVL is manufactured with 15 layers of 3 mm thick Douglas-fir veneers using Phenol-Formaldehyde-based resins resulting in a density of 573 kg/m^3^. The tentative elastic modulus of the LVL bar is 2.1 × 10^6^ lbs/in^2^ (14.47 GPa). The dimensions of each sample are 14.73 mm by 14.73 mm by 44.45 mm. A peculiar aspect of the experiment was the different orientations of the samples: three sample configurations were arranged parallel to each of the three orthogonal axes (longitudinal or L, tangential or T, and radial or R direction), whereas the other three sample configurations were arranged with an angle of 45° relative to each plane of the axes [13], as shown in Figure 2a. For each sample configuration, we tested three samples to understand the level of variance resulting in a total number of 18 test samples. 

The first objective of this compression test was to ascertain the maximum load the samples could endure within a linear region. Owing to the orthotropic nature of the layered engineered wood, it became critical to test each sample for its permissible load capacity. The linear region, within this context, refers to the specific segment where the relationship between stress and strain remains linear, and the resulting deformation is predominantly elastic. Furthermore, the engineering elastic constants were obtained through measuring sample responses to uniaxial compressive loads via strain gauges. Three strain gauges were affixed for the three sample configurations oriented along the principal axes (L, T, and R). One was placed longitudinally along the length, and the other two were positioned transversely along the width on the opposing faces of the initial strain gauge [13]. These gauges were vital for registering the strain in their respective directions. Strain measurements obtained played a crucial role in calculating critical mechanical properties, including Young’s modulus and Poisson’s ratio. The other three samples oriented at a 45° angle were instrumented with two strain gauges: one along the length and the other along the width, on opposing faces. Strain measurements obtained from these gauges were used to determine the shear modulus in each of the three planes. Strain gauges used for the experiment are the uniaxial strain gauges (Micro Measurement C4a-060SL-39P-350, Micro-Measurements, Raleigh, NC, USA) with a gauge factor of 2.09. The strain gauge arrangement is shown in Figure 2b,c. Strain values that can be extracted from each sample are listed in Table 1. *ε*_L_, *ε*_T_, and *ε*_R_ are strain values recorded by strain gauges aligned with the L, T, and R directions, respectively. These values were used to calculate elastic moduli and Poisson’s ratios. *ε*_H_ and *ε*_V_ are the horizonal and vertical strain values for the 45°-inclined S_4_, S_5_, and S_6_. These values were then converted to the shear strain which was used to compute the shear moduli. 

The loading process was facilitated by an Instron 5969 Dual Column Testing System (Instron, Norwood, MA, USA) with a maximum capacity of 50 kN, as shown in Figure 2c. The Instron load frame was configured to apply a uniaxial compressive load through a constant displacement rate of 0.01 mm/min and used its built-in load cell for force measurements. Throughout the compression test, the load and displacement were continuously recorded. The strain gauge readings were acquired using a VPG 40 strain gauge data acquisition system (Micro-Measurements, Raleigh, NC, USA) linked to a computer. This system enabled real-time data streaming and storage, and the strain data were further processed to ascertain strain values in length and width directions.

To uphold the integrity and reliability of the results, three samples were tested on each sample configuration. The average values of strains and forces obtained from these repetitions were utilized to compute engineering constants following Equation (1) [13], including three moduli of elasticity ($E_{L}$, $E_{R}$, and $E_{T}$), three shear moduli ($G_{LR}$, $G_{LT}$, and $G_{RT}$), and six Poisson’s ratios ($\nu_{LR}$, $\nu_{RL}$, $\nu_{LT}$, $\nu_{TL}$, $\nu_{RT}$, and $\nu_{TR}$). Once the engineering constants are obtained, the 6 × 6 stiffness matrix can be obtained based on Equation (2), where the principal axes 1, 2, and 3 align with L, R, and T, respectively.

(1) $$\begin{array}{c} {S1:E}_{T}=\frac{\sigma_{T}}{\varepsilon_{T}}\;\;\;\;\;\nu_{TL}=\frac{\varepsilon_{L}}{\varepsilon_{T}}\;\;\;\;\;\nu_{TR}=\frac{\varepsilon_{R}}{\varepsilon_{T}}\\ {S2:E}_{R}=\frac{\sigma_{R}}{\varepsilon_{R}}\;\;\;\;\;\nu_{RL}=\frac{\varepsilon_{L}}{\varepsilon_{R}}\;\;\;\;\;\nu_{RT}=\frac{\varepsilon_{T}}{\varepsilon_{R}}\\ {S3:E}_{L}=\frac{\sigma_{L}}{\varepsilon_{L}}\;\;\;\;\;\nu_{LR}=\frac{\varepsilon_{R}}{\varepsilon_{L}}\;\;\;\;\;\nu_{LT}=\frac{\varepsilon_{T}}{\varepsilon_{L}}\\ S4:G_{LT}=\frac{\tau_{LT}}{\gamma_{LT}}\;\;\;\;\;\;\;\;\;\;\;\;\;\;\;\;\;\;\;\;\;\;\;\;\;\;\;\;\;\;\;\;\;\;\;\;\;\;\\ {S5:G}_{LR}=\frac{\tau_{LR}}{\gamma_{LR}}\;\;\;\;\;\;\;\;\;\;\;\;\;\;\;\;\;\;\;\;\;\;\;\;\;\;\;\;\;\;\;\;\;\;\;\;\;\\ {S6:G}_{RT}=\frac{\tau_{RT}}{\gamma_{RT}}\;\;\;\;\;\;\;\;\;\;\;\;\;\;\;\;\;\;\;\;\;\;\;\;\;\;\;\;\;\;\;\;\;\;\;\;\; \end{array}$$

(2) $$\left[\begin{array}{cccccc} C_{11} & C_{12} & C_{13} & 0 & 0 & 0\\ C_{21} & C_{22} & C_{23} & 0 & 0 & 0\\ C_{31} & C_{32} & C_{33} & 0 & 0 & 0\\ 0 & 0 & 0 & C_{44} & 0 & 0\\ 0 & 0 & 0 & 0 & C_{55} & 0\\ 0 & 0 & 0 & 0 & 0 & C_{66} \end{array}\right]=\left[\begin{array}{cccccc} \frac{E_{L}}{1-\nu_{LR}\nu_{RL}} & \frac{E_{R}\nu_{RL}}{1-\nu_{LR}\nu_{RL}} & \frac{E_{T}\nu_{TL}}{1-\nu_{LT}\nu_{TL}} & 0 & 0 & 0\\ \frac{E_{R}\nu_{RL}}{1-\nu_{LR}\nu_{RL}} & \frac{E_{R}}{1-\nu_{RT}\nu_{TR}} & \frac{E_{T}\nu_{TR}}{1-\nu_{TR}\nu_{RT}} & 0 & 0 & 0\\ \frac{E_{T}\nu_{TL}}{1-\nu_{LT}\nu_{TL}} & \frac{E_{T}\nu_{TR}}{1-\nu_{TR}\nu_{RT}} & \frac{E_{T}}{1-\nu_{TL}\nu_{LT}} & 0 & 0 & 0\\ 0 & 0 & 0 & G_{RT} & 0 & 0\\ 0 & 0 & 0 & 0 & G_{TL} & 0\\ 0 & 0 & 0 & 0 & 0 & G_{LR} \end{array}\right]$$

### 2.2. Guided Wave Measurements

**Figure 3:**
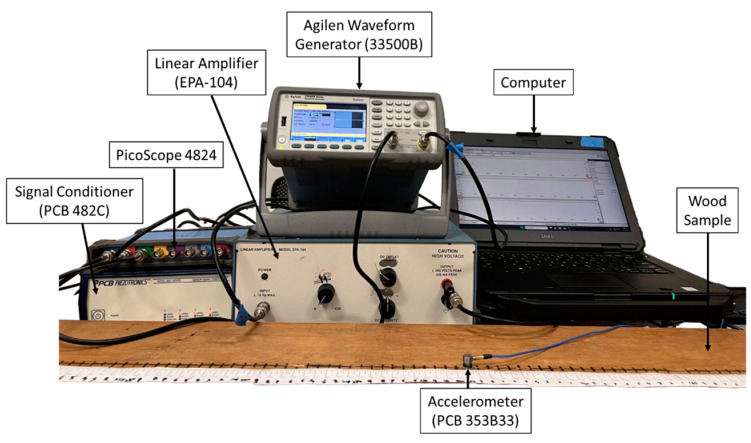
Experimental setup for guided wave measurements.

An LVL bar from the same manufacturer and the same model number with a length of 2500 mm and a cross-section of 241.3 mm by 44.45 mm was used for the guided wave measurements. Both ends of the LVL bar were wrapped with clays to minimize wave reflections. A Lead Zirconate Titanate (PZT) disc (Navy type II, APC International, Ltd., Mackeyville, PA, USA) with a diameter of 14 mm and thickness of 12 mm was attached to the top surface of the LVL bar close to one end. A chirp voltage excitation swept from 1 to 12 kHz with an amplitude of 10 Volts Peak to Peak (Vpp) was configured via an Agilent 33500B series waveform generator (Agilent Technologies, Santa Clara, CA, USA). The chirp signal was set as 0.3 s long, amplified 20 times using an EPA-104 Linear Amplifier (Piezo Systems, Inc., Woburn, MA, USA), and applied on the PZT disc. Data collection was performed every 10 mm with a total of 200 measurement points. The out-of-plane wave motions were recorded at each data collection point using a uniaxial accelerometer (PCB model 353B33, PCB Piezotronics, Depew, NY, USA), which were first passed through a signal conditioner (PCB 482C, PCB Piezotronics, Depew, NY, USA) and then digitized by the Pico Scope 4824 (pico Tchnology, St. Neots, Cambridgeshire, UK) with a 1 MHz sampling frequency, as shown in Figure 3. Ten repetitive data collections were conducted and averaged for each point to improve the signal-to-noise ratio (SNR). The obtained temporal–spatial sampling of dynamic responses was then converted to dispersion relations in the frequency–wavenumber domain via 2D-FFT using the MATLAB signal processing toolbox.

### 2.3. Numerical Models

Two types of finite element models were set up with the geometry’s cross-section consistent with the experimental sample to compute dispersion curves and structural dynamic responses. First, the Floquet periodicity was adopted for dispersion curve calculation. Floquet periodicity refers to the periodic behavior observed in the response of a material under certain conditions [39,40]. This theorem applies broadly to various branches of physics and engineering, including quantum mechanics, optics, and acoustics. In the study of periodic structures, like photonic crystals, Floquet’s theorem allows us to take advantage of the repeating nature of the structure to reduce a seemingly infinite problem into a manageable one. In the context of wave propagation in continuum waveguides, Floquet theory is commonly used to analyze the dispersion relation as a mathematical function that gives the relationship between wavenumber and frequency. Floquet’s theorem can be applied to the wave equation describing the system.

(3) $$u_{dst}=u_{src}e^{-ik_{j}\left(r_{dst}-r_{src}\right)}$$

where *u*_dst_ and *u*_src_ are the displacements in the destination and source plane, *k_j_* is the *j*th wavenumber, and *r*_dst_ − *r*_src_ is the distance from the destination and source plane. By seeking solutions of the form of a plane wave modulated by a periodic function (a “Floquet mode”), relationships between frequencies and wavenumbers, namely, dispersion curves, were obtained, which provide essential information about how waves of different frequencies behave when they encounter continuum waveguide structures.

For the Floquet periodicity model, the extruded length was adjusted to near the periodic cell length for the Floquet periodicity. The wavenumber was then assigned to the wave propagation direction, and the eigenfrequencies were computed for each wavenumber. This gives us the wavenumber–frequency pairs for the designated search area, which was determined based on experimental observations. This model was then used to obtain the simulated dispersion curves for optimization. The material parameters were set as the ones obtained from the 1st compression test. 

**Figure 4:**
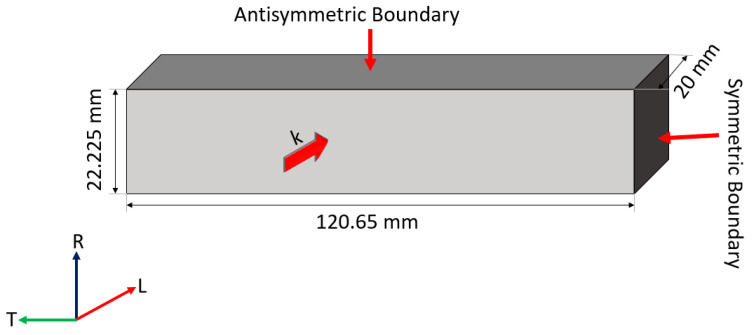
The quarter Floquet periodicity model.

Two Floquet periodicity models were established: a full model and a quarter model. The full model incorporates the full cross-section of 241.3 mm by 44.45 mm, whereas the latter one only needs to model a quarter of the cross-section using symmetric settings. As vertical flexural (VF) modes generally feature large out-of-plane motions, a symmetric boundary in the LR plane and an antisymmetric boundary in the LT plane were implemented in the quarter model, as shown in Figure 4. One of the cross-section faces is treated as the source and the other as the destination for sweeping the wavenumber (k) using the Floquet theorem. The unit cell thickness is set to 20 mm to ensure it can cover a reasonable range of wavenumbers for modes of interest. The mesh size is adjusted to 20 times smaller than the wavelength for model convergence.

**Figure 5:**
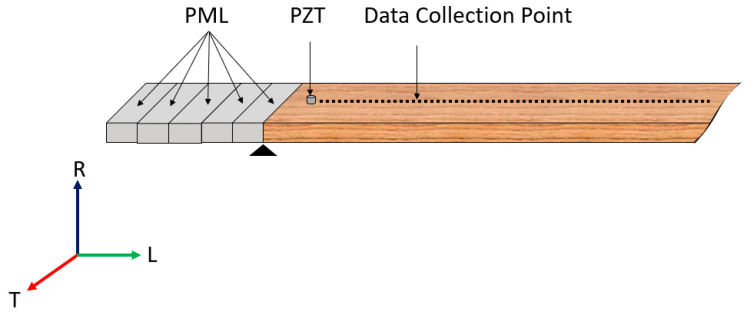
Frequency-domain model setup.

On the other hand, a frequency-domain numerical model was built to analyze wave modes promoted in the LVL bar by a PZT disc, which would be beneficial for optimization. The frequency-domain simulation was based on material properties from the 1st compression test and the exact dimensions as used in the experiment, along with the nominal piezoelectric material (PZT 5A equivalent to APC 850 Navy II material, APC International, Ltd., Mackeyville, PA, USA). The PZT disc was modeled using the Electrostatics module in COMSOL combined with the Solid Mechanics module. These two components were then coupled using the piezoelectric effect Multiphysics module. A 20 Volt excitation was applied to the terminal of the PZT disc where the other end was grounded. As shown in Figure 5, five layers of Perfectly Matched Layer (PML) domains were added to both ends of the model to suppress the reflection of the waves [41], which simulates the suppression of wave reflection in the experimental setup using clays. The mesh size was set up to be 7.5 mm, which is approximately 1/20 of the wavelength for the given input excitation. The frequency was then swept from 2 to 7.5 kHz, which is the targeted frequency range. Steady-state solutions at 200 points with a 10 mm interval on the top surface were obtained for harmonic excitation at each frequency. Complex displacements sampled through the data collection points were converted to dispersion relations in the frequency–wavenumber (f–k) domain using FFT. 

### 2.4. Parameter Identification Using Genetic Algorithm

**Figure 6:**
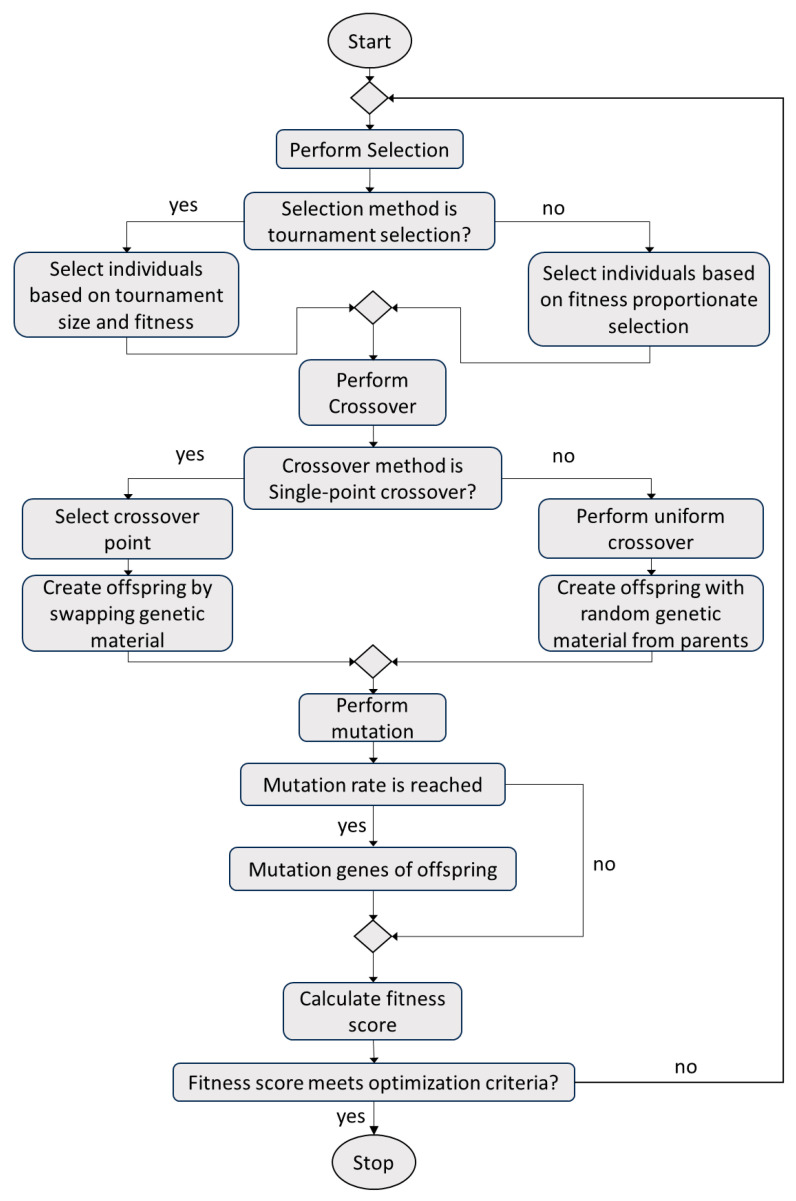
Flowchart of genetic algorithm (GA).

An effective method for material parameter identification is utilizing guided waves and corresponding dispersion curves. Dispersion curves represent the relationships between the frequency and wavenumber of guided modes and are sensitive to variations in material parameters and geometry. Thus, a genetic algorithm (GA) was proposed for identifying mechanical properties by updating the Floquet periodicity model until it yields dispersion curves closely resembling experimental dispersion relations. The general workflow for the genetic algorithm is shown in Figure 6. In general, mechanical properties are first fed into the GA to be optimized and randomized, resulting in a set of N populations. It is notable that population is defined as a set of individuals at a particular iteration of the genetic algorithm, and an individual is the group of parameters to be optimized, namely, the elastic constants from C_11_ to C_66_. The fitness function is then calculated for each set of parameters and returns a fitness value that signifies if it is the best result (lower value being better). Based on the fitness value, the population is selected for the next generation, similar to biological evolution. The rank selection process selects random individuals based on their rank, which was used in conjunction with tournament selection. The selected population then undergoes crossover, where specific population parameters become exchanged. Similarly, there is a chance of mutation, where some of the populations are again randomized. Mutation was conducted to introduce randomness during the selection process, and adjusting the mutation rate could allow for the algorithm to not become stuck in local minima and achieve the global optimization. Thus, after these steps, a new set of offspring populations is formed, and the process continues for several generations, which controls the maximum number of iterations as well as the model convergence. The genetic algorithm is a multi-variable global optimization technique, which is ideal for the problem at hand.

Specifically, initial parameters based on the elastic constants from the compression tests were assigned with a search region ranging from 0.1 to 2 times the initial values. The population size was defined as 48, and the number of generations was 100. The mutation rate was set as 10. Rank selection was adopted in conjunction with BLX-alpha crossover. The rank selection process selects the populations based on the ranking instead of assigning the populations a value based on their fitness value, which helps select a broader range. The BLX-alpha crossover operator performs a linear combination of the parent solutions’ genes, allowing for exploration and exploitation of the search space. The gamma parameter within this crossover operator controls the extent of exploration. The fitness function compares dispersion curves from Floquet periodicity models with the experimental dispersion relations, where a polynomial fit was performed on multiple branches of the experimental dispersion relations. Each point’s (eigenfrequency and wavenumber pair) distance to all the polynomial curves was calculated, and the closest curve was chosen for the point. The fitness value is defined as the sum of the squared distance of the points from their associated curves divided by the total number of points.

(4) $$Fitness\;Value=\frac{1}{N}\sum_{i=1}^{N}{\left(y\left(x_{j},p\right)-y_{j}\right)}^{2}$$

where *x*_i_ is the *j*th frequency, *y_j_* is the corresponding wavenumber value, *p* is the polynomial coefficient of the closest curve and *N* is the total number of frequency-wavenumber pairs. 

**Figure 7:**
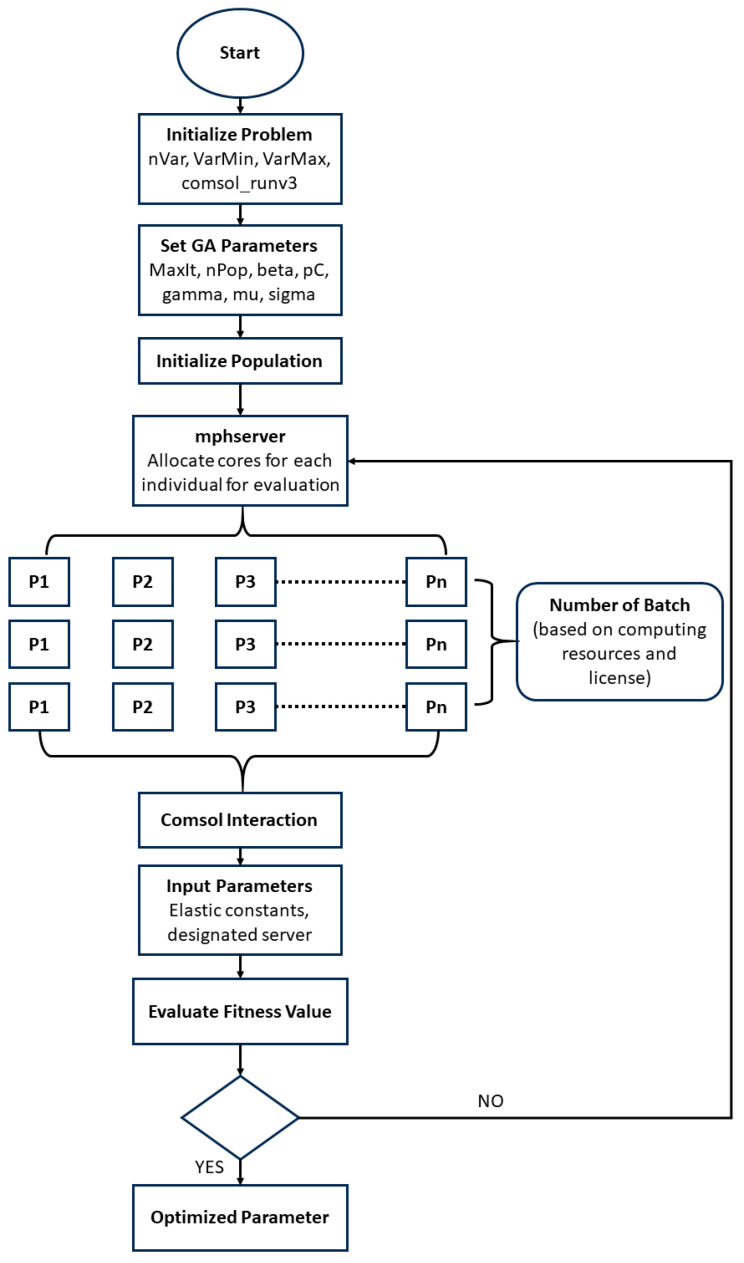
Algorithm implementation via LiveLink interaction between COMSOL and MATLAB at the CHPC at the University of Utah.

To handle the computation complexity, the numerical model and GA were implemented on designated cores in the Center for High-Performance Computing (CHPC) at the University of Utah through a workflow shown in Figure 7. With the COMSOL MATLAB LiveLink protocol, for each batch of population from P_1_ to P_n_, multiple COMSOL servers were assigned based on the available cores at the CHPC. Hyperparameters, including MaxIt, nPop, beta, pC, and mu, control the maximum number of iterations, the number of populations, probability distribution function during selection, probability of crossover, mutation rate, and standard deviation that determines the extent of mutation. Servers performed parallel COMSOL simulations for each individual model (which comprises a set of optimizable parameters). The limit to the number of iterations would be set based on the change in the fitness value over the iterations or the maximum of 100. Inputs (elastic constants) for all COMSOL models were configured via MATLAB R2023b, and the estimated dispersion curves were extracted and compared with the ground truth to compute the fitness value. The optimization was conducted in two rounds due to the time allocation limitations on the CHPC servers. This process helped to limit the search area for the simulation as well as lead to more accurate parameter identification. As the optimization techniques were finalized, a sensitivity analysis was carried out to assess how the fitness value changes with varying mechanical properties.

## 3. Results and Discussion

### 3.1. Static Compression Test

**Figure 8:**
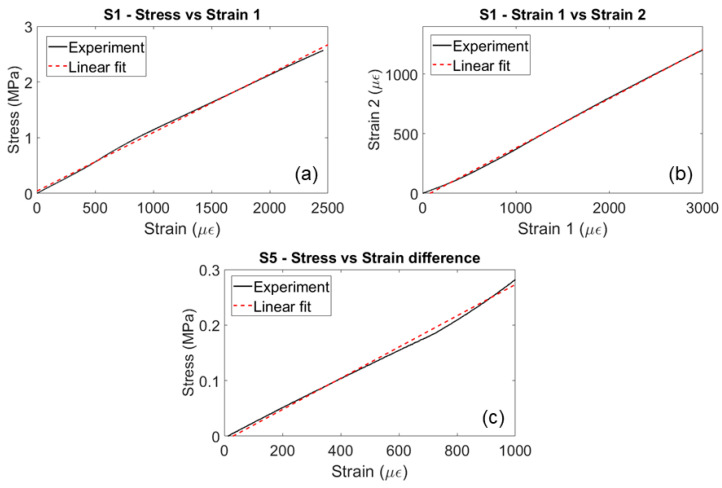
Stress–strain and strain–strain curves for determining engineering elastic constants.

The stress–strain curves from the compression tests were obtained for each sample to calculate elastic constants and Poisson’s ratios. According to Table 1 and Equation (1), elastic moduli can be calculated by using the load cell for stress calculation and strain gauges for strain measurements. Ratios of stress and strain yield the moduli of elasticity, whereas the ratio of strains at various directions gives Poisson’s ratios. The linear fit of the stress–strain and strain–strain curves from S1 and S5 is showcased in Figure 8. The stress–strain curve fits linearly and yields Young’s modulus for samples S1, S2, and S3, which are the samples aligned with the T, R, and L directions, respectively. Poisson’s ratios were estimated based on strain–strain curves of these samples. Similarly, for the three angled samples (S4, S5, and S6), the ratio of the stress and the difference in strains gives three shear moduli of elasticity. This results in the evaluation of all the nine independent elastic constants for the LVL material. Results from three compression tests are shown in Table 2. The variations in material parameters through these compression tests are significant and can be attributed to internal defects and natural variability within the material, as the engineered wood is made from natural wood veneers where defects internal to and between veneers, as well as knots and disbonds, can affect the wood samples’ performance. 

It is notable that compression tests can characterize material properties and be used as initial guesses for optimizing parameter values. However, the coefficient of variance or CoV and standard deviation illustrate that there are significant variations in test results due to the natural fiber orientation, defects, and dissimilarities within samples. Our observations are consistent with the high CoV in engineering elastic constants of solid wood materials reported by the existing literature [13,14] and confirmed that compression tests are not the best method to acquire elastic constants. The compression test results were compared with approximate elastic constants from general softwood, including Douglas fir, in Table 3. The values for softwood were summarized based on compression tests performed on multiple natural wood types [37], so they were only used as a rough reference in comparison to the LVL samples. The values of elastic modulus ratios and Poisson’s ratio from softwood and LVL generally agree well, except on the occasion of *G*_RT_/*E*_L_. This could result from the manufacturing of LVL, as the RT plane has layers of wood sheets stacked using adhesive. Its material properties differ significantly from natural wood, resulting in such a discrepancy. Similarly, the Poisson’s ratio varies from the softwood Poisson’s ratio because of the addition of the adhesive layer and the manufacturing process. The engineering elastic constants obtained from compression tests were converted to elastic constants through Equation (2), which were then used as the initial guess for optimization. 

### 3.2. Sensitivity Analysis and Performance Verification

**Figure 9:**
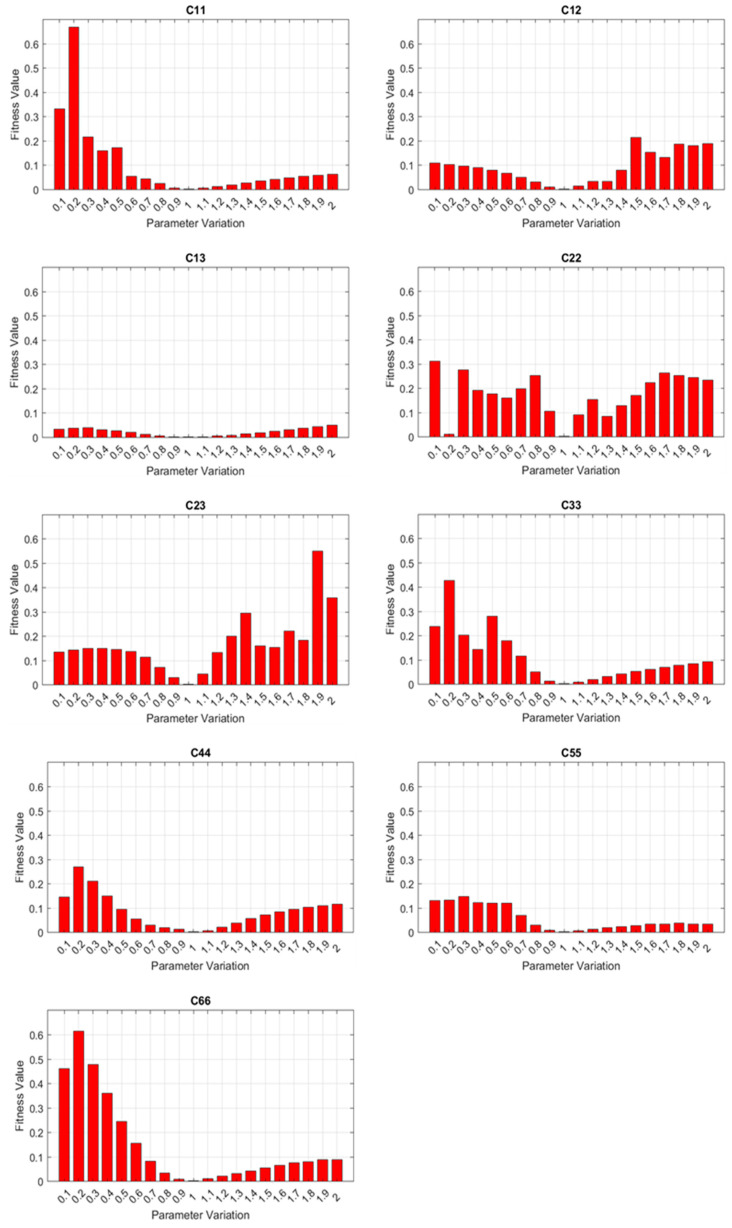
Sensitivity analysis based on the full Floquet periodicity model.

Prior to performing parameter identification, parametric sensitivities were analyzed to assess if changes in material parameters would induce sensible variations in dispersion curves. A parametric sweeping was performed on each of the nine elastic constants obtained from the first compression test. For each elastic constant, we swept its value from 0.1 to 2 times the corresponding nominal value, where other parameters were set as nominal values. The fitness values were computed based on Equation (4) for parametric sweeping. Because the fitness values were computed against the ground truth based on the set of nominal values, nominal elastic constants shall yield the minimum error or fitness value. The sensitivity analysis results based on the full Floquet periodicity model, where all the wave modes were considered, are shown in Figure 9. It is notable that a full set of wave modes from the full Floquet periodicity model can support reasonable sensitivity to the majority of material parameters, except *C*_13_ and *C*_55_. Variations in fitness values throughout the parametric sweeping indicate the sensitivity: the larger the change in amplitude, the more sensitive the dispersion curves are to the specific parameter. Moreover, the presence of local minimums in the sensitivity study could also affect the performance. 

**Figure 10:**
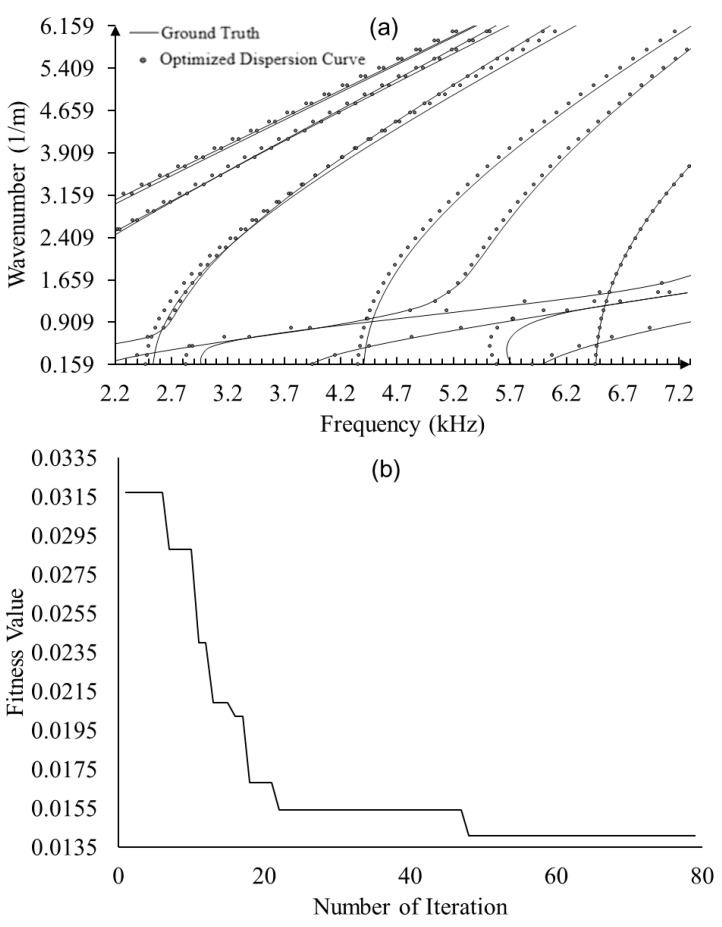
Optimization results based on full Floquet periodicity model: (**a**) comparison of optimized dispersion curve with ground truth; and (**b**) fitness values through generations in GA.

To validate the proposed framework, optimization was performed using dispersion curves predicted by the full Floquet periodicity model with the nominal material properties as the ground truth to estimate the mechanical properties. As shown in Figure 10a, the optimized elastic constants yield dispersion curves (dots) resembling the ground truth (solid lines)—dispersion curves based on nominal mechanical properties. Moreover, the fitness value converged to 0.0135 around 80 generations, as shown in Figure 10b. The estimated elastic constants and errors are shown in Table 4, yielding an excellent estimation accuracy except for *C*_13_ and *C*_55_, for which the full wave modes are less sensitive according to the sensitivity analysis.

**Figure 11:**
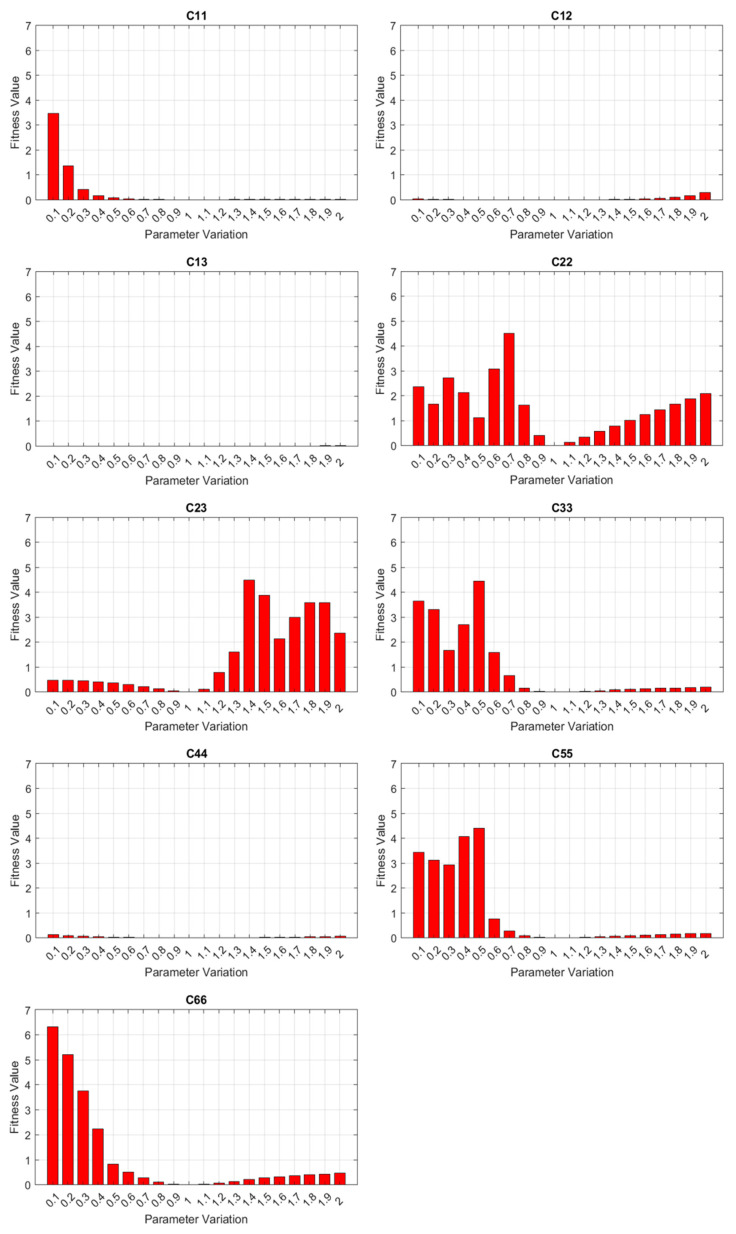
Sensitivity analysis based on the quarter Floquet periodicity model.

**Figure 12:**
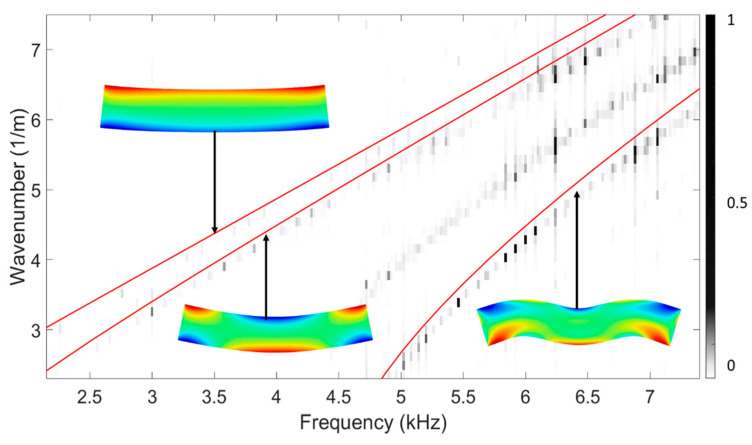
Dispersion relations from frequency-domain simulation overlaid with dispersion curves generated from the quarter Floquet periodicity model (red lines).

However, not all wave mode families can be easily excited in practice. The vertical flexural or VF mode family was used in our experiment due to its easier-to-measure out-of-plane components compared to other mode families. Thus, sensitivity analysis was performed on all the elastic constants only using dispersion curves of the VF mode family, in which *C*_11_, *C*_12_, *C*_22_, *C*_23_, *C*_33_, *C*_55_, and *C*_66_ demonstrate appreciable sensitivities measured by the fitness function, as shown in Figure 11. Moreover, frequency-domain finite element analysis was conducted to obtain dispersion relations when the LVL bar is subjected to PZT’s excitation. While it generates both VF and longitudinal modes, VF modes are generally dominant in experimental measurements due to the test setup. With nominal material properties and consistent waveguide geometry, the dispersion relations align well with the dispersion curves from the quarter Floquet periodicity model, as shown in Figure 12. It is notable that the frequency-domain analysis agrees with the result of the quarter Floquet periodicity model except for one mode, which was not captured by the quarter Floquet theorem-based model. The mode shapes based on the eigenanalysis demonstrate an antisymmetric displacement field in terms of displacement along the wave propagation direction, which verifies the excited vertical flexural modes.

**Figure 13:**
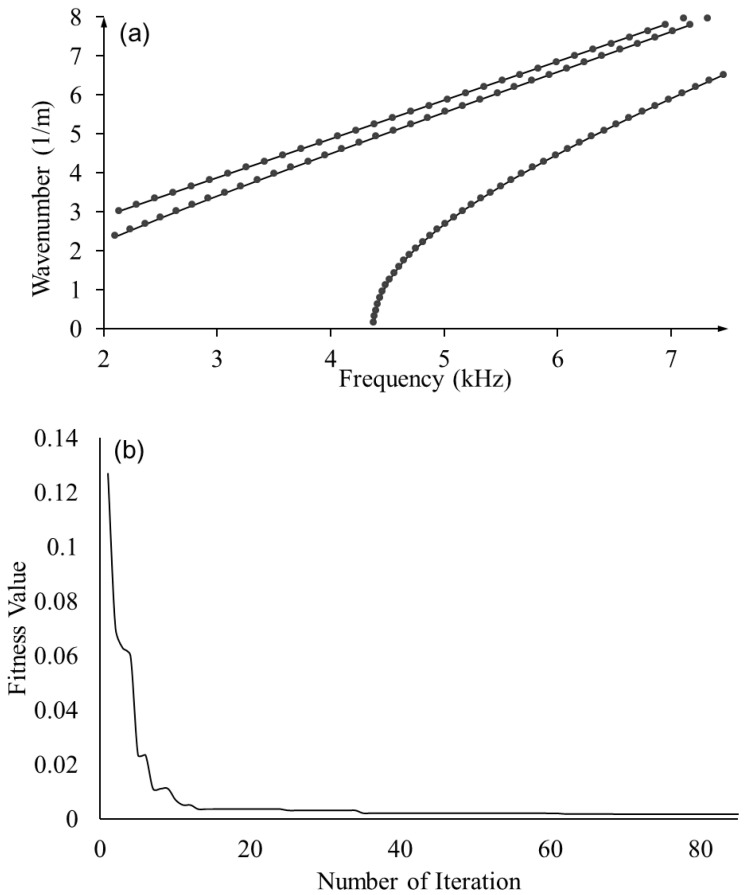
Optimization results based on quarter Floquet periodicity model and frequency-domain simulation: (**a**) comparison of optimized dispersion curve with ground truth (ground truth: solid lines; optimization results: dots); and (**b**) fitness values through generations in GA.

By extracting polynomial fitted curves from the simulated dispersion relations, optimization was performed using three vertical flexural modes only. A reasonable agreement was obtained between dispersion curves generated by the quarter Floquet periodicity model using nominal values (ground truth) and simulated dispersion relations from the frequency-domain analysis, as shown in Figure 13a. The parameters converged in 82 generations with a fitness score of 0.0105, as shown in Figure 13b. Due to the lack of sensitivities to a subset of elastic constants, estimated material parameters, as shown in Table 5, demonstrate significantly higher error ratios compared to the full model prediction (Table 4). The elastic constants of *C*_13_, *C*_23_, and *C*_55_ demonstrate errors over 15%. According to Figure 11, the vertical flexural modes show little sensitivity to *C*_13_ and low sensitivity in a wide range of *C*_23_ and *C*_55_. 

### 3.3. Optimization Based on Experimental Guided Wave Measurements

Upon the verification of the proposed framework using finite element models, experimental dispersion relations (vertical flexural wave modes only) and the quarter Floquet periodicity model were used to identify elastic constants of the LVL bar. The sensitivity analysis in Figure 11 shows that not all material parameters can be successfully optimized using the vertical flexural wave mode family alone, and our effort of optimization based on frequency-domain analysis has resulted in a reasonable estimation of parameters, including *C*_11_, *C*_12_, *C*_22_, *C*_33_, *C*_44_, and *C*_66_. 

**Figure 14:**
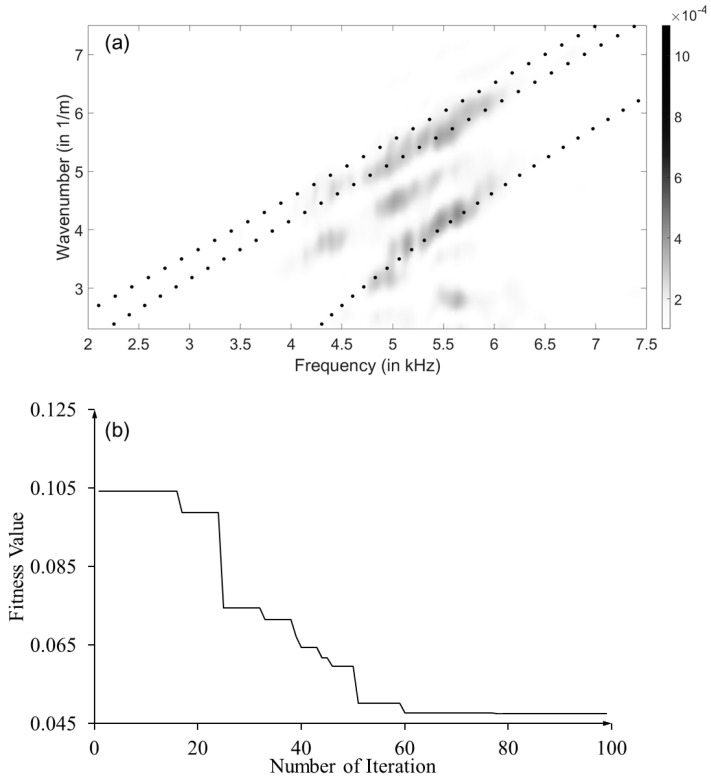
Optimization results based on the quarter Floquet periodicity model and experiments: (**a**) comparison of optimized dispersion curves overlaid with experimental dispersion relations (experimental dispersion relations: color map; optimized dispersion curves: dots); and (**b**) fitness values through generations in GA.

While previous researchers demonstrated difficulties in performing guided wave measurements in layered engineered wood [22], the experimental dispersion relations exhibit clear multi-modal wave propagation behavior in the LVL bar, as shown in Figure 14a. There are noises caused by the intrinsic heterogeneity of the LVL waveguide. Similar to the frequency-domain analysis, an additional mode between vertical flexural branches was observed, which was not captured by the quarter Floquet periodicity model. The polynomial fits from the experimental dispersion relations were extracted and fed to the optimization process. The optimization was performed by using averaged elastic constants from the compression test as the initial values. The optimization results generally agree with our experimental observations. As shown in Figure 14b, the optimization process converges after 100 generations, resulting in a fitness score of 0.047. The prediction performance is summarized in Table 6. While initial values are elastic constants obtained from three compression tests, these values are also limited by available sample sizes and shall be considered as references rather than ground truth. As expected from the sensitivity analysis, *C*_13_ and *C*_55_ have consistently large discrepancies from the initial values due to the lack of sensitivity from specific wave modes. In addition, *C*_11_ was also observed to have a significant discrepancy, which could be attributed to the uncertainties from compression tests. 

## 4. Conclusions

This study presented the first comprehensive investigation of material characterization for orthotropic layered engineered wood structures using multi-modal guided waves and the genetic algorithm. The laminated veneer lumber, or LVL, derived from natural solid wood, is susceptible to internal defects and delamination, making it imperative to comprehend their material properties for potential NDE and quality assurance of engineered wood components and structures. Our investigation encompassed both destructive compression tests and nondestructive guided wave measurements on LVL samples, with the aim of inferring orthotropic material properties. To achieve this, a sophisticated multi-parameter optimization technique, namely the genetic algorithm, was developed, which was tailored to determine the elastic constants of engineered wood by carefully defining an appropriate fitness function. The sensitivity analysis of dispersion curves to variations in material properties was systematically examined, leading to the precise definition of a fitness function capable of quantifying prediction errors. By minimizing fitness values, a set of optimized material parameters that closely matched the dispersion curves were derived based on actual material properties. Further performance evaluation using a larger sample size of compressive testing is needed to ensure the accuracy and reliability of the optimization process.

Our current study primarily focused on the determination of material properties of layered engineered woods, employing an inversion method to optimize the nine sets of independent material properties to best fit the dispersion curves obtained from guided waves. The experimental setup, however, was designed to excite vertical flexural modes, prompting us to optimize parameters sensitive to these modes. While the specific mode family lacks sensitivity to some of the elastic constants, our study successfully optimized a subset of the nine elastic constants. It is essential to note that our objective was to develop a method employing guided waves and the GA optimization tool for estimating material properties, which need to undergo further verification before their application in the structural design process. Future research endeavors can extend the optimization scope to include not only the elastic constants but also the viscoelastic properties of engineered wood. In our study, the low-frequency spectrum of guided waves was studied, which are typically less prone to attenuation. However, it is essential to account for the viscoelastic effect inherent in wood characteristics. The elasticity matrix, composed of elastic constants, is intricate, and incorporating viscoelasticity into the optimization technique can facilitate the optimization of the attenuation coefficient of engineered wood. While the feasibility of the proposed framework has been verified in this study, statistical modeling and analysis on the framework’s performance is necessary to make informed decisions for NDE and QA purposes. This holistic approach would enable a more comprehensive characterization of the material, dispelling the assumption of negligible viscoelastic effects and thus enhancing the accuracy of material property determination for practical applications in structural design and engineering.

## Figures and Tables

**Table 1:** Strain gauges and their corresponding strain values for each sample.

S1	Strain Gauge 1: *ε*_T_	Strain Gauge 2: *ε*_L_	Strain Gauge 3: *ε*_R_
S2	Strain Gauge 1: *ε*_R_	Strain Gauge 2: *ε*_L_	Strain Gauge 3: *ε*_T_
S3	Strain Gauge 1: *ε*_L_	Strain Gauge 2: *ε*_R_	Strain Gauge 3: *ε*_T_
S4	Strain Gauge 1: *ε*_V_	Strain Gauge 2: *ε*_H_	
S5	Strain Gauge 1: *ε*_V_	Strain Gauge 2: *ε*_H_	
S6	Strain Gauge 1: *ε*_V_	Strain Gauge 2: *ε*_H_	

**Table 2:** Engineering elastic constants based on strain gauge measurements.

Engineering Constants	Test 1	Test 2	Test 3	Average	Standard Deviation	CoV
*E*_L_ (GPa)	18.20	13.30	9.79	13.77	3.45	25.1%
*E*_R_ (GPa)	0.99	1.30	0.92	1.07	0.16	15.3%
*E*_T_ (GPa)	0.85	0.89	0.90	0.88	0.02	2.5%
*ν*_TL_	0.049	0.041	0.047	0.05	0.003	7.4%
*ν*_TR_	0.599	0.719	0.712	0.677	0.055	8.1%
*ν*_RL_	0.023	0.046	0.003	0.024	0.018	73.2%
*ν*_RT_	0.364	0.679	0.233	0.425	0.187	44.0%
*ν*_LR_	0.485	0.339	0.313	0.379	0.076	20.0%
*ν*_LT_	0.418	0.590	0.268	0.425	0.132	30.9%
*G*_LT_ (GPa)	1.64	0.68	0.87	1.06	0.42	39.0%
*G*_LR_ (GPa)	0.96	0.81	0.66	0.81	0.12	15.3%
*G*_RT_ (GPa)	1.33	1.10	0.30	0.91	0.44	48.6%

**Table 3:** Comparison between mechanical properties of softwood and LVL.

Ratio or Poisson’s Ratio	Softwood [15,31]	LVL Test Results
*E*_L_/*E*_R_	13	18.4
*E*_L_/*E*_T_	21	21.4
*G*_LR_/*E*_L_	15	16.5
*G*_LT_/*E*_L_	17	11.2
*G*_RT_/*E*_L_	153	13.7
*ν*_LR_	0.3	0.48
*ν*_RL_	0.003	0.01
*ν*_LT_	0.43	0.43
*ν*_TR_	0.31	0.60
*ν*_TL_	0.02	0.04
*ν*_RT_	0.51	0.36

**Table 4:** Optimized material parameters and corresponding errors utilizing full Floquet periodicity model.

Elastic Constants	Estimation (GPa)	Ground Truth (GPa)	Error (%)
*C*_11_	15.90	16.10	1.69
*C*_12_	2.30	2.37	3.13
*C*_13_	2.71	2.20	23.23
*C*_22_	1.82	1.83	0.44
*C*_23_	1.39	1.32	5.30
*C*_33_	3.02	3.20	5.45
*C*_44_	0.55	0.54	2.39
*C*_55_	0.96	0.78	22.79
*C*_66_	0.60	0.62	2.38

**Table 5:** Optimized material parameters and corresponding errors utilizing vertical flexural modes.

Elastic Constants	Estimation (GPa)	Ground Truth (GPa)	Error (%)
*C*_11_	14.90	16.10	7.39
*C*_12_	2.16	2.37	8.89
*C*_13_	1.72	2.20	21.85
*C*_22_	2.02	1.83	10.68
*C*_23_	1.55	1.32	17.62
*C*_33_	2.98	3.20	6.87
*C*_44_	0.51	0.54	4.20
*C*_55_	0.92	0.78	17.35
*C*_66_	0.62	0.62	1.10

**Table 6:** Optimized material parameters and errors based on experimental dispersion curves.

Elastic Constants	Estimation (GPa)	Initial Values (GPa)	Difference (%)
*C*_11_	45.36	30.39	49.24
*C*_12_	3.11	3.24	3.83
*C*_13_	5.28	4.07	29.52
*C*_22_	1.87	1.87	0.40
*C*_23_	2.55	2.50	1.82
*C*_33_	5.65	5.48	3.11
*C*_44_	0.87	0.93	6.33
*C*_55_	2.30	1.49	54.15
*C*_66_	0.61	0.64	3.96

## Data Availability

The data presented in this study are available on request from the corresponding author.
